# Selling cannabidiol products in Canada: A framing analysis of advertising claims by online retailers

**DOI:** 10.1186/s12889-021-11282-x

**Published:** 2021-07-01

**Authors:** Marco Antonio Zenone, Jeremy Snyder, Valorie Crooks

**Affiliations:** 1 Faculty of Health Sciences, Simon Fraser University, Burnaby, BC V5A 1S6 Canada; 2 Department of Geography, Simon Fraser University, Burnaby, BC V5A 1S6 Canada

**Keywords:** Cannabidiol, CBD, Cannabis, Canada, Advertisement, Retail

## Abstract

**Background:**

In Canada, the legalization of cannabis has enabled cannabidiol (CBD) to become a popular commercial product, increasingly used for medical or therapeutic purposes. There are currently over one thousand CBD products available globally, ranging from oil extracts to CBD-infused beverages. Despite increased usage and availability, the evidence supporting the medical efficacy of CBD is limited. Anecdotal evidence suggests CBD sellers represent their products for medical use through direct medical claims or advice, which in Canada, is not allowed under the *Cannabis Act* without Health Canada approval. However, it is not clear the extent of sellers making health claims or other strategies used to promote medical usage of CBD. The objective of this study is to determine how CBD sellers advertise their products online to consumers.

**Methods:**

The product descriptions of 2165 CBD products from 70 websites selling CBD products for human consumption in Canada were collected from January 14th^,^ 2020 to February 2nd^,^ 2020 using an automated website scraper tool. A framing analysis was used to determine how CBD sellers frame their products to prospective customers. The specific medical conditions CBD is represented to treat and product forms were tabulated.

**Results:**

CBD products are framed to prospective customer through three distinct frames: a specific cure or treatment (*n* = 1153), a natural health product (*n* = 872), and a product used in certain ways to achieve particular results (*n* = 1388). Product descriptions contained medical or therapeutic claims for 171 medical conditions and ailments, with 53.3% of products containing at least one claim. The most prevalent claims found in product descriptions were the ability to treat or manage pain (*n* = 824), anxiety (*n* = 609), and inflammation (*n* = 545). Claims were found for treating or managing serious and-life-threatening illnesses such as multiple sclerosis (*n* = 210), arthritis (*n* = 179), cancer (*n* = 169), Crohn’s disease (*n* = 78), Parkinson’s disease (*n* = 59), and human immunodeficiency virus (HIV) (*n* = 54). CBD most often came in oil/tincture/concentrate form (*n* = 755), followed by edibles (*n* = 428), and vaporizer pen/cartridge/liquid products (*n* = 290).

**Conclusion:**

The findings suggest CBD is represented as a medical option for numerous conditions and ailments. We recommend Health Canada to conduct a systematic audit of companies selling CBD for regulatory adherence.

## Background

The popularity of cannabidiol (CBD) – one of many cannabinoids found in cannabis and hemp plants – has increased in Canada and globally in recent years [1]. By 2024, the CBD industry expects to reach a market valuation of 20 billion USD [2]. CBD products are diverse - they include cosmetic products such as makeup, hygiene products such as shampoos, edibles, beverages, oils, and vaporizer products such as infused vape pens or liquids.

Media sources often attribute CBD’s popularity to its representation as a medicinal cannabis product, capable of treating numerous conditions and ailments without the psychoactive effects of tetrahydrocannabinol (THC) [3–5]. The majority of medical cannabis users in Canada (41%) reported using products with higher CBD content over products with high THC [6]. CBD retailers and proponents commonly advertise CBD as helpful for uses such as inflammation, pain, epilepsy, depression, insomnia, anxiety, cancer, multiple sclerosis, skin health, and Alzheimer’s disease [3, 7–10]. Academics and journalists alike have pointed out that CBD is described as a ‘cure-all’ despite limited research confirming such general or specific claims [10–13].

At present, only one CBD-derived medication for rare seizure disorders – Epidiolex – is clinically accepted for treatment by the Food and Drug Administration (FDA) in the United States [14]. In Canada, there are three products containing cannabis approved for sale with drug identification numbers – Sativex, Marinol, and the synthetic cannabinoid Nabilone [15]. Marinol was voluntarily withdrawn from market. The supporting evidence for CBD medical uses besides seizure relief is promising but in the early stages of development [16–18].

Cannabis sellers, including sellers of CBD, are not allowed to make health or therapeutic claims in Canada or the United States. Under the *Cannabis Act*, the legislation governing cannabis products in Canada: “It is prohibited to promote cannabis, a cannabis accessory or a service related to cannabis … if there are reasonable grounds to believe that the promotion could create the impression that health or cosmetic benefits may be derived” [19]. The *Cannabis Act* also stipulates that cannabis cannot be promoted in a “manner that is false, misleading or deceptive or that is likely to create an erroneous impression about its characteristics, value, quantity, composition, strength, concentration, potency, purity, quality, merit, safety, health effects or health risks” [20]. Similarly, the United States prohibits the marketing of CBD products with unproven medical claims [21].

Despite the existence of regulations, there is evidence to suggest that sellers of CBD are representing their products as medical options for serious illnesses. In November of 2019, the FDA issued warnings to 15 companies making unproven claims of CBD efficacy for conditions such as cancer and Parkinson’s disease [22]. Similar observations are reported in Canada. For example, an unlicensed CBD brand known as ‘Mona Lisa Healing’ made claims during the COVID-19 pandemic that their CBD can “help your body defend against COVID-19 coronavirus” [23]. Marijuana Business Daily, a cannabis industry news source, acknowledges the medical representation of CBD products to prospective consumers: “Cannabis products with unauthorized health claims are commonly sold in unregulated channels, especially products containing CBD” [24]. It is unclear if such claims are widespread across the CBD industry or an outlier, and thus represent an area of needed research.

The present study contributes to filling this research gap by systematically exploring how CBD products are advertised for sale on Canadian cannabis retail websites. Approximately 20% of medical cannabis users in Canada order their products online [6]. Sellers of CBD typically seek to describe the benefit(s) of purchasing their product, and thus online product messaging is an ideal source to determine how retailers are representing CBD products to consumers. The exploration of online CBD product advertisements in Canada will contextualize industry adherence to existing Government of Canada regulations and inform public health policies.

## Methods

To identify CBD products available for sale online by Canadian retailers, two Google searches of ‘buy CBD Canada’ and ‘buy cannabidiol Canada’ were completed on January 14th^,^ 2020. The website URL on the first 20 pages of each search (400 URLs retrieved) was recorded. This broad search term phrasing mimics Google searches a prospective CBD consumer may use to identify and find information on CBD products. After identifying 400 websites that potentially sell CBD products, a duplicate filter left 296 websites for review to determine if they sold CBD to Canadian consumers.

For inclusion into the study, the websites retrieved must have sold at least one CBD product for human consumption/use and it must have been possible to purchase such products from the website directly, without a medical provider’s approval or license. The website operators must also have been based in Canada or individually operated a Canadian division and thus were subject to Canadian regulations. After implementing a review of each website, 68 websites met the inclusion criteria. During the review, seven websites selling CBD were identified as being run by a provincial or territorial government, inclusive of British Columbia, Alberta, Ontario, New Brunswick, Newfoundland and Labrador, Yukon, and Nova Scotia. We searched for other government-run online retailers in the other provinces or territories not identified in the original website search. After doing so, an additional two websites were added, from Prince Edward Island and the Northwest Territories, totaling 70 identified websites selling CBD in Canada. The remaining provinces and territories (Saskatchewan, Manitoba, and Nunavut) did not have a government-run CBD retail website.

The first author then retrieved each CBD product URL from the 70 included websites. We considered CBD products to be those that through naming or product description contained an equivalent or higher amount of CBD than THC. The URLs from each website were searched and collected through one of three ways: (1) reviewing all products included in CBD-specific sections of websites; (2) reviewing products identified from a ‘CBD’ and ‘cannabidiol’ keyword search in the search function of the website; or (3) in the absence of a search function or CBD section, hand searching all pages of a website to identify and review products. After the website reviews were completed, a total of 2165 CBD product URLs were recorded by website.

To collect product details, including product name, company, CBD form, product description, and price, two methods were used. The website and its associated URLs were either data scraped using custom made DataMiner codes (*n* = 34 websites) or the information manually retrieved through visiting the website and recording the above data fields (*n* = 36 websites). Decisions to use either method depended on (1) the number of URLs/product descriptions to extract by website, where generally, more than 15 products led to the development of a scraper code, or (2) if website contained protections where a scrape job could not be completed due to reasons such as a manual requirement to confirm the user was not a computer bot, age verification checks, or other formatting issues.

After collection, each author independently reviewed 10% of products and met to determine an appropriate analytic framework. After discussion, we agreed to use case study methodology where framing analysis - a qualitative analysis technique to examine how information (e.g., an advertisement, a new story) is framed to convey a particular belief – to explore how online CBD product retailers in Canada framed CBD product uses and benefits in their web advertisements [25, 26]. After further independent data review and another collaborative meeting we confirmed three dominant frames for CBD products, which was that they were: (1) a treatment or cure for specific ailments; (2) a natural health product; and (3) a product used in specific ways to achieve particular results. To support our exploration of the first and third frames, we also counted the specific medical conditions or medical applications suggested in the CBD product descriptions on the reviewed websites.

## Results

The websites identified (*n* = 70) had on average 30.9 (median, 21) CBD products for sale (see Table 1). The most popular form of CBD product available was oil/tincture/concentrate (*n* = 755), followed by edibles (*n* = 428), vaporizer pen/cartridge/liquid (*n* = 290), topical/cosmetic products (*n* = 200), capsules/pills/soft gels (*n* = 178), dried cannabis flower products (*n* = 163), bath products (*n* = 80), beverages (*n* = 36), patches (*n* = 21), assorted product (*n* = 7), suppositories (*n* = 5), and other forms (*n* = 2) (see Table 2).

**Table 1:** Number of CBD Products by Website and Framing Code

Website	Framing #1: Treatment or Cure	Framing #2: Natural Health Product	Framing #3: Specific Usage & Results	Total Products
herbapproach.com	72	46	85	118
cbd-oil-canada.ca	66	55	84	111
leaf2go.ca	31	21	85	109
thehealingco.ca	59	34	63	96
birchandfog.com	66	37	65	85
budlab.ca	76	51	60	81
justcannabis.shop	56	25	64	80
chilliwackcbd.ca	57	44	60	78
cbd2go.ca	52	55	55	75
shopcbdonline.ca	39	28	52	74
magnoliawellness.ca	53	34	23	73
buybudnow.ca	25	41	46	71
speedgreens.ca	14	10	24	53
mjnexpress.ca	27	22	28	46
cannabis-nb.com	0	7	12	44
bccannabisstores.com	0	6	22	43
albertacannabis.org	0	6	10	42
weedsmart.ca	2	1	11	40
topshelfexpress.ca	12	11	25	40
buymyweedonline.ca	37	12	37	38
motacannabisproducts.ca	35	5	27	37
mailorder-marijuana.ca	27	8	30	36
canadablissherbals.com	24	18	25	34
happybears.ca	26	28	29	32
ocs.ca	0	7	15	29
sqdc.ca	8	19	9	29
shopcannabisnl.com	0	0	0	28
buycbdcanada.ca	19	15	17	27
delta9.ca	0	1	8	27
buymellow.com	12	22	14	24
feelcbd.ca	18	21	23	24
getkush.ca	12	15	13	24
greensociety.ca	17	10	19	23
cannabiscare.ca	11	11	17	22
cannabis.shoppersdrugmart.ca	22	0	22	22
headz.ca	8	7	14	20
lowcloud.ca	10	7	8	20
ganjaexpress.ca	12	1	10	19
westcoastsupply.ca	12	6	11	18
canadaonlinethc.ca	3	1	3	18
balancecbd.com	1	15	17	17
thefoggyforest.ca	13	2	3	16
platinumherbalcare.com	14	6	9	16
high420.ca	2	7	8	15
tokyosmoke.com	0	2	6	15
canadacannabisdispensary.ca	5	7	8	13
peicannabiscorp.com	0	5	2	12
cannabisdispensary.ca	11	3	7	12
greencanadacbd.ca	9	8	9	11
budsandbeyond.ca	6	1	5	9
firstwonder.ca	9	2	9	9
cannabisyukon.org	1	2	0	9
cannacanine.ca	8	1	1	8
bestpotdelivery.ca	7	6	8	8
vitalityhealthcbd.com	3	6	8	8
greenleafexpress.ca	2	8	8	8
earthchoicesupply.com	8	8	8	8
calyxwellness.co	6	5	6	6
honestbotanicals.ca	4	5	6	6
zenabis.com	0	0	5	6
houseofcannabis.ca	2	0	6	6
cannawholesalers.ca	4	2	4	6
canadianbotanicaldrops.ecwid.com	6	6	6	6
icaria.co	1	5	5	6
bcweedpen.com	4	0	0	4
cbddirectonline.ca	1	4	3	4
cbdme.store	1	4	1	4
hemp-canada.ca	3	2	3	3
okanagancbd.com	2	2	2	2
doseofcanna.ca	0	0	0	2
**Total**	**1153**	**872**	**1388**	**2165**

**Table 2:** Number of CBD Products by Form

Form	Frequency
Oil / Tincture / Concentrate Products	755
Edibles	428
Vape pen / cartridge / liquid / kit	290
Topical / cosmetic	200
Capsules / pills / softgels	178
Dried cannabis flower products	163
Bath products	80
Beverages	36
Patch	21
Assorted	7
Suppositories	5
Other	2
**Total**	**2165**

### Frame #1: treatment or cure for specific ailments

The first framing theme identified, *treatment or cure for specific ailments* (*n* = 1153 products), contained product claims of suggestive efficacy and effectiveness for 171 medical conditions, ailments or symptoms (Table 3 summarizes claims with a minimum frequency of 20 claims). Treatment and curative claims ranged from minor ailments such as pain, inflammation, or bruising, to severe ailments such as replacement of conventional cancer treatment, Parkinson’s disease treatment, and neurological conditions. For example, a dried flower product from the bestpotdelivery.ca website stated their product was “very effective for chronic medical conditions like bipolar disorder, chronic stress, depression, panic attacks, anxiety disorders, and post-traumatic stress disorder” and that “one lungful and you’ll feel all your depression and stress melt away.” Other CBD product descriptions contained long lists of purported treatments and conditions. For example, a product description for a CBD infused lollipop from herbapproach.com listed the following purported uses: “Medical Conditions [treated by CBD]: ADD, ADHD, Anxiety, Appetite, Arthritis, Asthma, Back Pain, Bipolar Disorder, Body Pain, Cachexia, Cancer, Cramps, Crohn’s Disease, Depression, Epilepsy, Fibromyalgia, Gastrointestinal Disorder, Glaucoma, Headaches, Hepatitis C, HIV/Aids, IBS, Inflammation, Insomnia.”

**Table 3:** Number of Medical Claims by Condition/Symptom (minimum frequency, *n* = 20)

Conditions	Frequency
Pain	824
Anxiety	609
Inflammation	545
Stress	386
Sleep	316
Muscle relief	236
Depression	229
Multiple sclerosis (MS)	210
Insomnia	200
Appetite stimulant	190
Arthritis	179
Nausea	170
Cancer	169
Spastic-symptoms	154
Epilepsy	136
Mood	136
Unspecified ailments/conditions/diseases	135
Neurologic health	106
Skin health	98
Post-traumatic stress disorder (PTSD)	96
Seizure relief	95
Neuropathy	79
Crohn’s disease	78
Migraines	74
Irritable Bowel-Syndrome	64
Joint relief	63
Attention hyperactivity disorder (ADHD)	61
Fibromyalgia	61
Parkinson’s disease	59
Eczema	55
Attention deficit disorder (ADD)	54
Headache	54
Human immunodeficiency virus (HIV)	54
Acne	52
Acquired immunodeficiency syndrome (AIDS)	52
Hepatitis C	52
psychotic	51
Alzheimer’s	50
Psoriasis	49
Immune function	48
Gastrointestinal health	47
Energy (fatigue relief)	44
Neuroprotective	43
Colitis	42
Diabetes	42
Sexual health	41
Spinal cord	40
Dizziness	39
Eating disorders	39
Brain injuries	38
Muscular dystrophy	38
Analgesia	37
Nervous system health	37
Cramp	35
Menstrual health	34
Glaucoma	28
Vomiting	26
Addiction	25
Aging	25
Cell damage and/or regeneration	25
Circulation	25
Discomfort	25
Mobility	24
Cardiovascular health	23
Mental clarity	23
Rash relief	22
Scarring	21

To support CBD as a treatment for the numerous conditions or ailments collected in the product descriptions, retailers or the CBD companies provided references to published research studies or used anecdotal feedback from their customers or, in some cases, themselves. When describing the research, retailers often used animal-based studies or study designs that were not suitable for clinical adoption to support their claims. For example, a CBD capsule product from thehealingco.ca stated: “A rarely discussed health benefit of CBD oil is how it can reduce the risk of developing diabetes. In a study … 32% of the mice that received the CBD were diagnosed with diabetes, compared to 100% of the untreated group.” The details of the studies, including information on study limitations, were often not disclosed. For example, a CBD infused vaporizer liquid from thefoggyforest.ca stated: “A lot of studies have been done that point to CBD being beneficial for fighting cancer. Less potent cancer cells were found in breast tumours when a person uses CBD.” The product description further states: “[T]here’s also one study that shows dependent cell death thanks to the CBD oil. This non-toxic compound, when used at 700 mg per day for six weeks, showed no signs of toxicity.”

Testimonials for product efficacy were referenced in some product descriptions. Statements of anecdotal effectiveness did not make general claims, but rather detailed how they had been helpful for certain people and relieved their symptoms or condition. For example, a product description from a CBD Move gummy product on the healingco.ca website contained anecdotal feedback from the company founder and his social network. He described how his experience with CBD had motivated him to bring the benefits to others: “After an injury, the creator of CBD Move discovered the power of CBD. As he says himself [he] started recommending CBD to friends and relatives suffering from different physical ailments, arthritis, headaches etc. Every single time CBD provided them ease and welcome relief.” The framing of *CBD as a treatment or cure for specific ailments* was represented through direct claims, seller interpretation of CBD research, and through anecdotal product testimonials.

### Frame #2: natural health product

The second framing identified, *a natural health product* (*n* = 872 products), sold the natural benefits of CBD and its advantage over conventional health products. Most often, products described CBD as a natural option to treat pain. For example, a CBDfx lotion product sold on the chilliwackcbd.ca website stated: “This skin-nourishing cream lets you target your pain ‘hot spots’ with the healing power of 150 mg of full-spectrum CBD hemp oil and other natural pain relievers.” Products in numerous cases advocated using CBD in place of other conventional medications or treatments. These ranged from minor substitutions, such as using CBD for inflammation, to suggestions of potential treatment replacement of severe illnesses. For example: “Over the past few decades, informed consumers have begun a major shift away from synthetic pharmaceutical drugs with their long lists of debilitating side effects, in favour of health-enhancing natural remedies” and that “CBD, from hemp is providing that all-natural solution for many, and word of its success at providing an amazing variety of therapeutic benefits for a host of physical and mental disorders.”

Natural framing of CBD commonly described the cultivation methods and additional ingredients or precautions used to formulate the product. Natural health product terms such as “organic”, “pure”, “non-GMO”, “pesticide-free”, and “naturally grown” were viewed in product descriptions as selling qualities. For instance, an edible CBD infused gummy product from balancecbd.com made the following statement to support the role of organically produced products on health: “Our tasty gummies are made with the finest organic ingredients, without any animal by-products, cementing our commitment to providing a natural way to enjoy the benefits of CBD.” Another tincture product from earthchoicessupply.com used a similar method of selling the organic production of their product and its effect on health: “try our 3000mg Premium Hemp Tincture Oil if you want a quick and effective way of getting all the benefits offered by this unique plant. Natural and Organic are the best words to describe our 30ml Tincture Oil that contains 3000 MG CBD Oil and 0% of THC!” CBD was framed as *a natural health product* through descriptions of natural benefits, cultivation methods, or ingredients. The natural characteristics of CBD were used by sellers to illustrate its claimed advantages over conventional products and terminology associated with natural products emphasized for safety.

### Frame #3: a product used in specific ways to achieve particular results

The third frame, *a product used in specific ways to achieve particular results* (*n* = 1388), utilized characteristics of treatments to describe CBD products and advertise them to potential buyers. These included describing CBD products in terms of treatment unit quantities, labelling the use of CBD products as treatment, offering comparison to medical or pharmaceutical standards, and identifying treatment methods of administration. CBD quantities were characterized as “doses” and contained language using precise amounts to describe CBD concentration. These statements were similar to pharmaceutical advice and direction. For example, a CBD gummy product from happybears.ca stated that “[e] ach chewy gummy combines a concentrated dose of pure CBD with the natural calming properties of Melatonin to promote restful sleep and morning wakefulness.” Product descriptions contained language that referenced the person taking CBD as receiving “treatment” or being a “patient” for using a CBD product. For example, a zenabis.com CBD spray stated the intended user of their product was a patient: “Each 0.1 ml spray (at full compression) of Zenabis’ High CBD 30:0 Spray offers patients 3 mg of CBD and 0 mg of THC in Medium-chain Triglyceride (MCT) oil.”

Comparisons to existing standards of conventional treatment were observed in these campaigns. Products utilized such terminology such as “pharmaceutical grade” or “medical grade” to support the potential health impact of product use. For example, a CBD product from CBD-EEZ on the birachandfog.com website that is sold in powder form stated: “each effervescent packet comes with 50mg of 99.9% pharmaceutical grade CBD to give you easy relief from anxiety, pain, insomnia and/or stress.” Similarly, the recommended intake method of CBD emulated conventional treatments. CBD product administration methods included ingestion, sublingual intake, topically, through capsules, pills, soft gels, syringes, suppositories and droppers. For example, an Emerald Health product on the Ontario Cannabis Store website (ocs.ca) stated: “This flavourless concentrated cannabis oil comes in a 20-mL bottle which includes a precision 1mL dosage syringe.” Sellers framed CBD as *a product used in specific ways to achieve particular results* by equating CBD to conventional forms of medications and treatment.

## Discussion

In Canada, the *Cannabis Act* restricts CBD sellers from making any suggestion or claim of efficacy for medical conditions without Health Canada authorization [15, 19]. Nonetheless, the majority of CBD products collected in our sample (53.3%) made at least one health claim, confirming the speculation of widespread CBD efficacy claims in contravention of the Cannabis Act [27, 28]. The top claims – that CBD treats or manages pain (*n* = 824), anxiety (*n* = 609), and inflammation (*n* = 545) – have been reported on significantly in the media [11, 29, 30]. The range of health uses recorded specifically included relief for minor symptoms such as stress (*n* = 386), disordered sleep (*n* = 316), muscle relief (*n* = 236), and nausea (*n* = 170). Purported health uses also covered treatment or symptom relief for chronic or life-threatening illnesses such as multiple sclerosis (*n* = 210), arthritis (*n* = 179), cancer (*n* = 169), Parkinson’s disease (*n* = 59), and Alzheimer’s diseases (*n* = 50). Taken together, these claims demonstrated the multiple uses for which CBD products were advertised and sold to Canadian buyers.

Product descriptions commonly framed CBD as a legitimate medicinal product with unique benefits. Sellers represented CBD as a cure or treatment for specific ailments and equated efficacy in the same terms as pharmaceutical products, in some cases advocating for the replacement of conventional products (for example, for pain relief). Misinterpretation of CBD research and overvaluation of efficacy testimonials lent credence to the seller’s assertions that such classification is correct and appropriate. Specific cure or treatment claims were supplemented by language for administering CBD products in specific ways to achieve particular results. This included information and instructions on methods of administration (oils/tincture droppers, capsules, syringes, and suppositories), product measurements and quantities (doses), and treatment protocols. Such instructions may have been used to reassure users of the product’s safety and effectiveness. Legitimacy was further established through stressing CBD’s natural benefits. These natural characteristics served to represent CBD as safer and more effective compared to pharmaceutical products.

Sellers referenced clinical studies and other sources of evidence to support the claims made in product descriptions, but the inaccuracies suggested overvaluation of and confusion regarding extant evidence of CBD’s efficacy as a medical treatment. The majority of sellers acknowledged the limited evidence available regarding CBD but framed it as a symptom of a new product with potential medical uses. Through this representation, sellers then presented the current evidence base as sufficient to demonstrate the plausibility of CBD for specific uses. Unsupported claims pose a concern for those using CBD for medical purposes, who may accept the accuracy of such claims and spend significant amounts of money on these products for potentially little benefit [31–33]. This is especially concerning in situations when CBD is purchased to improve or treat life-threatening illnesses among patients who may forego evidence-based treatments [34–36]. The spread of unproven and, in many cases, false claims, contributes to the spread of misinformation.

In response to the findings of the study, it is recommended that Health Canada conduct a systematic inspection of cannabis retailers selling CBD products for adherence to the *Cannabis Act*. The widespread CBD claims made by online retailers based in Canada or selling specifically to Canadian consumers make clear that non-compliance with existing regulations is prevalent, thus demonstrating a need to inspect CBD products to minimize consumer harm. Current risks to consumers buying CBD products under this context include unnecessary financial expenditure, purchasing based on false or unproven claims, and the spread of misinformation [34, 37, 38]. The authority available to Health Canada to ensure compliance under the *Cannabis Act* includes inspections [39]. Therefore, this is an appropriate intervention. Health Canada has the ability to issue cautions against those found not to be in compliance, including CBD retailers. These cautions can come in the form of warnings, fines, and additional inspections.

Further research on CBD in the Canadian context is needed. First, there is a need to investigate adherence to other *Cannabis Act* regulations, specifically, plain packaging and non-marketing of cannabis products to children. Many products included in the sample did not adhere to plain packaging restrictions. Labelling concerns are noted in the United States [40]. Products that appear to appeal to children, such as those mimicking popular candy bars or child-like characters, were identified and require further attention. Second, the role of in-person retail sellers requires analysis. The present study was limited to online products and thus cannot capture how retail employees or salespersons frame CBD to prospective consumers and how this may impact their decision-making.

The study has several limitations. First, the search strategy likely did not identify every website selling CBD online in Canada. We lessened this by using multiple approaches, and the intent of the study was not to identify every single website selling CBD. Second, a single investigator reviewed the content and assigned it to the relevant frame, this limitation was mitigated by using a triangulated approach to troubleshooting.

## Conclusion

Online Canadian retailers of CBD typically present products to potential customers through three distinct frames: a treatment or cure for specific ailments, a natural health product, and a product used in specific ways to achieve specific results. The framing of CBD lends support to the seller’s perception of CBD as a legitimate option for health purposes, despite a limited, and often misrepresented, evidence base. Online sellers of CBD in Canada demonstrate that many CBD products are not compliant with the *Cannabis Act* – with the majority of products in our sample making at least one health claim. Unproven claims have concerning implications for persons experiencing a medical illness or displaying symptoms, through unnecessary financial expenditure and the ineffectiveness of the product bought. The frames identified also contribute to the spread of CBD misinformation. Regulatory action is needed to monitor and inspect CBD sellers and ensure they comply with the *Cannabis Act*.

## Data Availability

The datasets used and/or analysed during the current study are available from the corresponding author on reasonable request.

## Abbreviations

CBD: Cannabidiol
THC: Tetrahydrocannabinol
FDA: Food and Drug Administration

## References

[1] Davis M. What Is CBD? Live Science. Available from: https://www.livescience.com/65811-what-is-cbd.html. Cited 2021 May 31

[2] Dorbian I. CBD Market Could Reach $20 Billion By 2024, Says New Study. Forbes. 2019 May 20. Cited 2021 May 31.

[3] Williams A. Why Is CBD Everywhere? The New York Times. 2018 Oct 27. Cited 2021 May 31.

[4] CBD products: favorite aspects in U.S. and abroad 2017. Statista. 2017 Dec 7. Available from: https://www.statista.com/statistics/789176/cbd-products-favorite-aspects-in-us-and-abroad/. Cited 2021 May 31.

[5] Walker LA, Koturbash I, Kingston R, ElSohly MA, Yates CR, Gurley BJ (2020). Cannabidiol (CBD) in dietary supplements: perspectives on science, safety, and potential regulatory approaches. J Diet Suppl.

[6] Canadian Cannabis Survey 2019 - Summary. Health Canada. 2019. Available from: https://www.canada.ca/en/health-canada/services/publications/drugs-health-products/canadian-cannabis-survey-2019-summary.html. Cited 2021 May 31

[7] MacKeen D. What Are the Benefits of CBD? The New York Times. 2019 Oct 16. Cited 2021 May 31.

[8] Velasquez-Manoff M. Can CBD Really Do All That? The New York Times. 2019. May 14 Cited 2021 May 31.

[9] Rabin RC. CBD Is Everywhere, but Scientists Still Don’t Know Much About It. The New York Times 2019 Feb 25. Cited 2021 May 31.

[10] Hsu T. Ads Pitching CBD as a Cure-All Are Everywhere. Oversight Hasn’t Kept Up. The New York Times. 2019 Aug 13. Cited 2021 May 31.

[11] CBD oil as health treatment: We answer 5 questions about cannabidiol products. CBC. 2019. Cited 2021 May 31.

[12] Caulfield T. CBD oil products promise miracle cures. But does science support the hype? NBC News. 2019 Mar 17. Cited 2021 May 31.

[13] Goodman S, Wadsworth E, Schauer G, Hammond D. Use and Perceptions of Cannabidiol Products in Canada and in the United States. Cannabis Cannabinoid Res. 2020. In Press. 10.1089/can.2020.0093. 10.1089/can.2020.0093 PMC9225398 33998872

[14] FDA Approves First Drug Comprised of an Active Ingredient Derived from Marijuana to Treat Rare, Severe Forms of Epilepsy. United States Food and Drug Administration. 2020 Mar 27. Available from: https://www.fda.gov/news-events/press-announcements/fda-approves-first-drug-comprised-active-ingredient-derived-marijuana-treat-rare-severe-forms. Cited 2021 May 31

[15] Health products containing cannabis or for use with cannabis: Guidance for the Cannabis Act, the Food and Drugs Act, and related regulations. Health Canada. 2018. Available from: https://www.canada.ca/en/health-canada/services/drugs-health-products/drug-products/applications-submissions/guidance-documents/guidance-cannabis-act-food-and-drugs-act-related-regulations/document.html. Cited 2021 May 31.

[16] Fiani B, Sarhadi KJ, Soula M, Zafar A, Quadri SA (2020). Current application of cannabidiol (CBD) in the management and treatment of neurological disorders. Neurol Sci.

[17] Khan R, Naveed S, Mian N, Fida A, Raafey MA, Aedma KK (2020). The therapeutic role of Cannabidiol in mental health: a systematic review. J Cannabis Res.

[18] Stanciu CN, Brunette MF, Teja N, Budney AJ (2021). Evidence for use of cannabinoids in mood disorders, anxiety disorders, and PTSD: a systematic review. Psychiatr Serv.

[19] The Cannabis Act and Cannabis regulations - Promotion prohibitions. Health Canada. 2018. Available from: https://www.canada.ca/en/health-canada/services/drugs-medication/cannabis/laws-regulations/promotion-prohibitions.html. Cited 2021 May 31.

[20] Consolidated federal laws of Canada, Cannabis Act. Justice Laws Canada. 2020. Available from: https://laws-lois.justice.gc.ca/eng/acts/c-24.5/page-4.html#h-77140. Cited 2021 May 31.

[21] What You Need to Know (And What We’re Working to Find Out) About Products Containing Cannabis or Cannabis-derived Compounds, Including CBD. United States Food and Drug Administration. 2021 Feb 8. Available from: https://www.fda.gov/consumers/consumer-updates/what-you-need-know-and-what-were-working-find-out-about-products-containing-cannabis-or-cannabis. Cited 2021 May 31.

[22] FDA warns 15 companies for illegally selling various products containing cannabidiol as agency details safety concerns. United States Food and Drug Administration. 2020. Available from: https://www.fda.gov/news-events/press-announcements/fda-warns-15-companies-illegally-selling-various-products-containing-cannabidiol-agency-details. Cited 2021 May 31

[23] Kronbauer B. Bif Naked launched an unlicensed CBD brand that claims it can help defend against Coronavirus. Vancouver Is Awesome. 2020 Mar 16. Cited 2021 May 31.

[24] Lamers M. Canada proposes ‘Cannabis Health Products’ category for humans and pets, paving way for large new market. Marijuana Business Daily. 2019 Jun 19. Cited 2021 May 31.

[25] Pan Z, Kosicki GM (1993). Framing analysis: an approach to news discourse. Polit Commun.

[26] Van Gorp B, Vercruysse T (2012). Frames and counter-frames giving meaning to dementia: a framing analysis of media content. Soc Sci Med.

[27] Subramaniam V. CBD-craze drives Canadian pot firms to hop on the hemp bandwagon. Financial Post. 2019 Feb 19. Cited 2021 May 31.

[28] Howatt B, Snider-Adler M. What you need to know about CBD and the workplace. The Globe and Mail. 2019 May 15. Cited 2021 May 31.

[29] Bedore C. CBD: Why people are turning to this popular pot product. Global News. 2019 Feb 22. Cited 2021 May 31.

[30] Young L. What you need to know about CBD, the non-intoxicating cannabis chemical. Global News. 2918 Dec 1. Cited 2021 May 31.

[31] Fleming A. Cannabis health products are everywhere – but do they live up to the hype? The Guardian. 2018. Cited 2021 May 31.

[32] Eisenstein M. The reality behind cannabidiol’s medical hype. Nature. 2019 Aug 28. Cited 2021 May 31.

[33] Zenone MA, Snyder J, Crooks VA (2021). What are the informational pathways that shape people’s use of cannabidiol for medical purposes?. J Cannabis Res.

[34] Stea JN, Marshall T. Cannabis: Misinformation about CBD can be life-threatening. The Conversation. 2019 Jul 9. Cited 2021 May 31.

[35] Zenone M, Snyder J, Caulfield T (2020). Crowdfunding Cannabidiol (CBD) for Cancer: hype and misinformation on GoFundMe. Am J Public Health.

[36] Leas EC, Hendrickson EM, Nobles AL, Todd R, Smith DM, Dredze M, Ayers JW (2020). Self-reported Cannabidiol (CBD) use for conditions with proven therapies. JAMA Netw Open.

[37] Greenberg S, Gremillion T. Buyer beware: False medical claims about CBD and COVID-19. The Hill. 2020 May 18. Cited 2021 May 31.

[38] White CM. No, CBD is not a miracle molecule that can cure coronavirus, just as it won’t cure many other maladies its proponents claim. The Conversation. 2020 Apr 15. Cited 2021 May 31.

[39] Compliance and enforcement policy for the Cannabis Act. Health Canada. 2018. Available from: https://www.canada.ca/en/health-canada/services/drugs-medication/cannabis/laws-regulations/compliance-enforcement-policy-cannabis-act/policy.html. Cited 2021 May 31.

[40] Corroon J, MacKay D, Dolphin W (2020). Labeling of Cannabidiol products: a public health perspective. Cannabis Cannabinoid Res.

